# Pt-Free Metal Nanocatalysts for the Oxygen Reduction Reaction Combining Experiment and Theory: An Overview

**DOI:** 10.3390/molecules26216689

**Published:** 2021-11-05

**Authors:** Heriberto Cruz-Martínez, Wilbert Guerra-Cabrera, Ernesto Flores-Rojas, Dunia Ruiz-Villalobos, Hugo Rojas-Chávez, Yesica A. Peña-Castañeda, Dora I. Medina

**Affiliations:** 1 Tecnológico Nacional de México, Instituto Tecnológico del Valle de Etla, Abasolo S/N, Barrio del Agua Buena, Santiago Suchilquitongo, Oaxaca 68230, Mexico; heri1234@hotmail.com (H.C.-M.); duniaruiz.itve.unionhgo@gmail.com (D.R.-V.); 2 Tecnológico Nacional de México, Instituto Tecnológico del Istmo, Panamericana 821, 2da., Juchitán de Zaragoza, Oaxaca 70000, Mexico; guerrawil@hotmail.com; 3 Instituto Politécnico Nacional, CICATA-Legaria, Legaria 694, Col. Irrigación, Ciudad de México 11500, Mexico; efloresr@cinvestav.mx; 4 Tecnológico Nacional de México, Instituto Tecnológico de Tláhuac II, Camino Real 625, Tláhuac, Ciudad de México 13508, Mexico; rojas_hugo@ittlahuac2.edu.mx; 5 Colegio de Ciencia y Tecnología, Universidad Autónoma de la Ciudad de México, Av. Fray Servando Teresa de Mier 92, Cuauhtémoc, Ciudad de México 06080, Mexico; 6 Tecnologico de Monterrey, School of Engineering and Sciences, Atizapan de Zaragoza 52926, Estado de Mexico, Mexico

**Keywords:** DFT calculations, electrochemical characterization, nanoparticles, PEM fuel cells, clusters

## Abstract

The design and manufacture of highly efficient nanocatalysts for the oxygen reduction reaction (ORR) is key to achieve the massive use of proton exchange membrane fuel cells. Up to date, Pt nanocatalysts are widely used for the ORR, but they have various disadvantages such as high cost, limited activity and partial stability. Therefore, different strategies have been implemented to eliminate or reduce the use of Pt in the nanocatalysts for the ORR. Among these, Pt-free metal nanocatalysts have received considerable relevance due to their good catalytic activity and slightly lower cost with respect to Pt. Consequently, nowadays, there are outstanding advances in the design of novel Pt-free metal nanocatalysts for the ORR. In this direction, combining experimental findings and theoretical insights is a low-cost methodology—in terms of both computational cost and laboratory resources—for the design of Pt-free metal nanocatalysts for the ORR in acid media. Therefore, coupled experimental and theoretical investigations are revised and discussed in detail in this review article.

## 1. Introduction

The proton exchange membrane fuel cells (PEMFCs) are electrochemical devices that have gained great importance because they directly convert the H_2_ chemical energy into electric energy. Such energy conversion occurs through two half reactions, namely the hydrogen oxidation reaction (HOR) and the oxygen reduction reaction (ORR) [1,2,3,4]. In addition, these devices present various other benefits. For example, they can be manufactured for different applications (e.g., electronic systems, vehicles and stationary power plants), they produce only water and heat as subproducts and they operate at low temperatures [5,6]. Despite all these advantages, there are still challenges that must be addressed before their large-scale commercialization. One of these challenges is related to the slow kinetics of the cathode reaction (ORR), which is various orders of magnitude slower than the HOR [7,8,9,10]. Consequently, the ORR limits the overall performance of the PEMFCs.

Currently, in order to solve the aforementioned drawbacks, commercial Pt/C nanocatalysts are used to accelerate the ORR kinetics in the PEMFCs devices. Nevertheless, this material has various disadvantages such as low availability, high cost and limited activity/durability [8,9]. That is why the design of novel nanocatalysts with high catalytic activity and stability as well as cost-effective production is very relevant to achieve large-scale commercialization of the PEMFCs [11,12,13,14,15]. So far, outstanding efforts have been developed to design more active, stable and cheaper nanocatalysts for the ORR in acid media. In the first instance, Pt-based nanocatalysts have been investigated, which have shown higher catalytic activity and stability than Pt/C [16,17,18,19,20,21,22]. Another route that has been utilized to obtain novel nanocatalysts for the ORR is the research of Pt-free metal nanocatalysts (e.g., Pd-, Ir-, Au- and Ag-based nanoparticles) [23,24,25]. The catalytic properties of these nanocatalysts are similar or superior to the Pt/C ones. Finally, carbon-based nanocatalysts (e.g., single-atom nanocatalysts, doped carbon, etc.) have been explored, since those present catalytic activities that are competitive or superior to Pt/C [26,27,28,29].

So far, at experimental level, nanocatalysts with controlled shape, size and composition have been synthesized, which have exhibited catalytic activities and stability much higher than commercial Pt/C [30,31,32,33]. For example, transition metal-doped (e.g., V, Cr, Mn, Fe, Co, Mo, W and Re) Pt-Ni octahedral nanoparticles were investigated for the ORR in acid media [31]. The undoped and doped Pt_3_Ni nanoparticles showed catalytic activities much higher than commercial Pt/C. For instance, the Mo-doped Pt_3_Ni/C nanocatalyst showed the best specific activity and mass activity, which were 81 and 73 times higher compared to the Pt/C nanocatalyst, respectively [31].

Along with experimental investigations, the substantial progress in the first principle or ab initio methods have made the investigation of novel computer-based nanocatalysts possible for the ORR [34,35]. Among the first principle methods, the density functional theory (DFT) investigations have been fundamental to understanding the electrochemical processes involved in the ORR at the atomic level [36,37]. The DFT computations have been used to explain, at the atomic level, the experimental evidence even more, and they have also been used as a predictive tool for the investigation of novel nanocatalysts for the ORR. Consequently, for the theoretical studies of ORR nanocatalysts, different catalytic activity predictors have been proposed, such as ORR intermediates’ adsorption energies, *d*-band center and free-energy diagrams of the ORR [38,39], which have shown good agreement with the experimental results. Therefore, these studies have motivated the theoretical investigation of novel nanocatalysts for the ORR in acid electrolytes [40,41,42,43,44,45].

In the last decades, to implement more active and stable nanocatalysts for the ORR, such nanocatalysts have been designed by combining experimental findings and theoretical insights. Given the great importance that the coupled of experimental and theoretical studies have acquired, a sizeable number of this kind of studies have been developed. Following this idea, a review article combining both approaches of research was published recently [39]. This article showed the advances in the design of Pt-based nanocatalysts for the ORR combining experiments and theory [39]. Currently, there are many investigations of this kind on Pt-free metal nanocatalysts. This is due to the importance that they have taken as substitutes of the Pt-based nanocatalysts for the ORR in acid media. However, there is no review articles on current developments of Pt-free metal nanocatalysts combining experiment and theory. Thus, in this review, advances in the design of novel Pt-free metal nanocatalysts combining experimental findings and theoretical insights are analyzed and discussed.

## 2. Pt-Free Monometallic Nanocatalysts

### Pd Nanocatalysts

Different novel metals have been investigated as substitutes of Pt-based nanocatalysts for the ORR in acid media. Among these, Pd has received considerable relevance due to its good catalytic activity, stability and a slightly lower than average price with respect to Pt in the last 10 years [24]. Accordingly, there are some coupled theoretical and experimental investigations of Pd nanocatalysts for the ORR in acid media [46,47,48,49]. It is well known that the shape of nanoparticles plays a very important role on the catalytic activity for the ORR. In this direction, Xiao et al. investigated the relationship between the morphology and the activity of the Pd nanocatalysts for the ORR [46]. They synthesized Pd nanorods and nanoparticles by modifying the precursors’ concentrations via the Pd electrochemical deposition. It was observed that the specific activity of Pd nanorods was 10-fold higher than that of Pd nanoparticles. Based on CO stripping, Pd nanorods have shown exposure on (110) facets of Pd. To understand the morphology and activity relationship of Pd nanocatalysts for the ORR, the oxygen adsorption energies (OAE) were investigated on different Pd facets. The OAE on Pd (110) is notably lower than that on Pd (100) and Pd (111). Thus, the superior activity of Pd nanorods for the ORR could be due to the exposure of Pd (110) facets [46].

On the other hand, other studies show that the compressive strain can decrease the adsorption energy of the reaction intermediates, thus favoring the catalytic activity for the ORR [50,51]. For this reason, Pd nanoparticles with compressive strains were investigated for the ORR [48]. For instance, Pd nanoparticles were synthesized via pulsed laser ablation in liquid with different NaCl concentrations. The size of the nanoparticles tended to decrease when the NaCl concentration increased. In such case, the lattice constant of nanoparticles synthesized using 0.01 M NaCl solution (L-Pd nanoparticles) was of 3.891 Å, while for the L-Pd nanoparticles annealed at 573 K for 2 h (A-Pd nanoparticles) and Pd/C, the constant was 4.004 and 4.015 Å, respectively, which showed the compressive strain of the L-Pd nanoparticles. Based on electrochemical tests, the catalytic activity of L-Pd nanocatalysts was much higher than that of A-Pd nanocatalysts, which can be attributed to the compressive strain of the L-Pd nanoparticles. In addition, the half-wave potential (E_1/2_) of L-Pd nanocatalysts was 0.78 V, which is very similar to that of Pt/C (E_1/2_ = 0.79 V). To corroborate the experimental evidence, Pd (111) surfaces with and without 3% strain were modeled (using an associative mechanism) to represent the Pd/C and L-Pd nanocatalysts, respectively. The Pd (111) surface with 3% compressive strain can weaken the adsorption of all intermediates; particularly, the OAE is reduced by 0.1 eV. This decrease in OAE can be directly associated with improved catalytic activity due to compressive strain [48].

Finally, Pd and B-doped (7 at. %) Pd nanoparticles supported on carbon were studied for the ORR combining experiment and theory [49]. At 0.90 V, the specific activity of the B-doped Pd nanocatalyst was 2.7 higher than Pd/C. To understand the origin of the enhanced catalytic activity of the B-doped Pd nanocatalyst, adsorption energies of O and OH on Pd (111) and Pd-B (111) surfaces were computed. It was demonstrated that the adsorption energy of O and OH on B-doped Pd was lower than on Pd surface. Based on theoretical and experimental evidence, the enhanced ORR catalytic activity could be attributed to the decrease of the adsorption energy of the reaction intermediates on B-doped Pd nanocatalyst compared with Pd nanocatalyst [49]. These results were consistent with other studies in which doping has been shown to be a good strategy to enhance the catalytic activity for the ORR [52,53].

## 3. Pt-Free Bimetallic Nanocatalysts

### 3.1. Pd-Based Bimetallic Nanocatalysts

One of the most used strategies to increase catalytic activity and reduce the cost of Pd-based nanocatalysts is the design of Pd-based bimetallic nanocatalysts, because they present catalytic activities superior to that of the Pd/C nanocatalysts [54,55,56,57,58]. In the first instance, the Pd-M/C (M = 3*d* metal) bimetallic nanoparticles are widely studied because 3*d* metals are cheap and abundant on the planet. In addition, it has been reported that Pd-M/C has higher catalytic properties than that of Pd/C [54,55,56,57,58]. Thus, there are many coupled theoretical and experimental investigations on Pd-M bimetallic nanocatalysts for the ORR in acid media.

One of the most studied systems combining experiment and theory is the Pd-Cu alloy [59,60,61,62,63]. It is well documented that the composition of the Pd-based alloys can have an important role on the catalytic activity for the ORR [64,65]. In this direction, there are coupled studies investigating the effect of the composition of the alloys on the catalytic activity for the ORR [59,60]. For instance, Savadogo and coworkers investigated the effect of alloying Cu with Pd as nanocatalyst for the ORR [59]. A change in the lattice parameters was observed when the content in the nanocatalyst of the Cu was modified. In addition, they determined that the best nanocatalyst is the Pd_50_Cu_50_ alloy. At theoretical level, they calculated the adsorption energies of molecular oxygen and the *d*-band center. In this study, they concluded that the *d*-band center decreased in relation to Pd metal, which produced an enhanced catalytic activity. Interestingly, when Zhong et al. studied Pd_n_Cu_100−n_ (n = 36, 54 and 75) alloys, they concluded the same finding [60]. Based on mass activity and specific activity measures, they agreed that the maximum catalytic activity for Pd-Cu alloys was reached when an atom ratio is close to 50:50. Besides, their theoretical results were similar to those reported previously in the literature [59].

**Figure 1:**
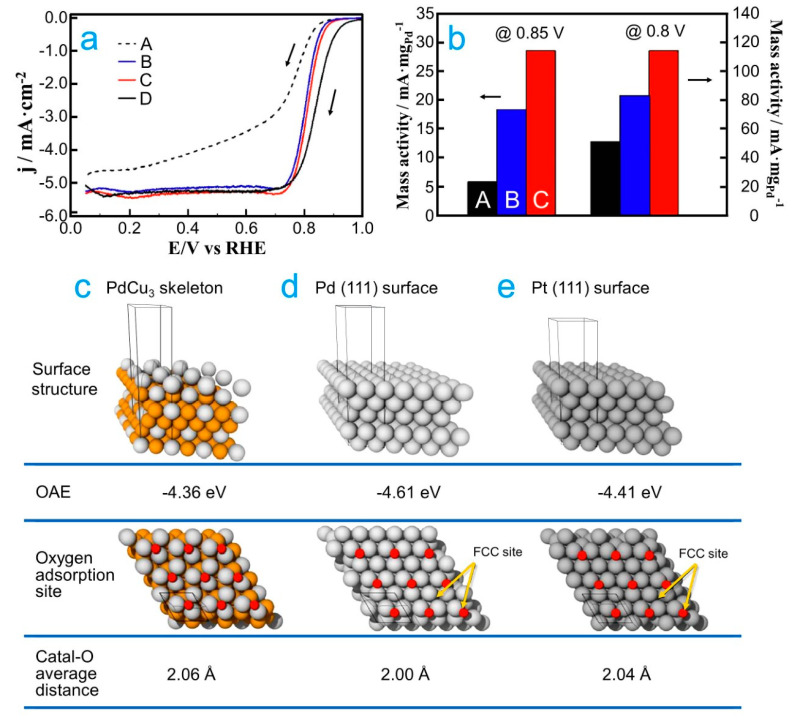
(**a**) Comparison of steady-state voltammograms for the ORR on (A) Pd NPs/CB, (B) Pd–Cu NPs/CB, (C) PdCu_3_ NPs/CB and (D) Pt NPs/CB in an O_2_-saturated 0.1 M aqueous solution of HClO_4_ at 5 mV s^−1^ and 1600 rpm. (**b**) Mass activity of each nanocatalyst (A, B and C) at 0.8 and 0.85 V. Oxygen binding energies, oxygen adsorption sites and average nanocatalyst surface–oxygen distances on the nanocatalysts: (**c**) electrochemically dealloyed PdCu_3_, (**d**) Pd (111) surface and (**e**) Pt (111) surface. Republished with permission of Royal Society of Chemistry from [62].

On the other hand, it has been observed that the structures of the alloys are very relevant on the catalytic activity [66,67]. In this way, Zhong and collaborators studied a PdCu nanocatalyst for the ORR, which was obtained by varying the thermal treatment conditions [61]. In this study, at 100 °C, the fcc structure appeared, whereas the fcc and bcc structures coexisted in the temperature range from 200 to 800 °C. Based on electrochemical procedures, the PdCu/C (H_2_/100 °C) nanocatalyst exhibited the higher catalytic activity. Notably, the PdCu/C nanocatalyst with the fcc structure showed a higher catalytic activity than the nanocatalysts with a mixture of fcc and bcc structures. To understand the correlation between the structure of the nanocatalysts and the catalytic activity for the ORR in acid media, DFT computations were carried out on fcc and bcc structures with PdCu (100) surfaces. The fcc PdCu alloy showed a lower reaction barrier to dissociate O_2_ than the bcc PdCu alloy. In addition, the relation between the electronic structure and catalytic activity of PdCu (100) and (111) alloys, with fcc structures, was investigated. Interestingly, a linear relationship was observed between the adsorption energies of atomic oxygen and the composition of the PdCu (100) and (111) alloys. In another study, Gunji and collaborators studied PdCu_3_-ordered intermetallic nanoparticles that were electrochemically dealloyed, which were supported on carbon black (PdCu_3_ NPs/CB) for the ORR [62]. Due to the electrochemical dealloying of PdCu_3_ NPs/CB, a Cu-free surface was obtained for this nanocatalyst. The PdCu_3_ NPs/CB nanocatalyst presented a higher catalytic activity than Pd/C and atomically disordered Pd–Cu nanoparticles supported on carbon black (Figure 1a,b). To understand the good catalytic activity of the nanocatalysts, *d*-band centers and the OAE were investigated. In that study, XPS profiles were obtained to estimate the *d*-band center values of the nanocatalysts. The *d*-band center of PdCu_3_ NPs/CB was −3.10 eV, which is lower than that of Pd NPs/CB (−2.96 eV), but similar to the calculated value for Pt NPs/CB (−3.19 eV). Based on DFT calculations, the OAE of PdCu_3_ (111) surface was lower than that on Pd (111) and Pt (111) surfaces (Figure 1c–e). Interestingly, using the OAE, DFT calculations showed that PdCu_3_ can have a higher catalytic activity than Pt for the ORR. However, the experimentally observed ORR activity for PdCu_3_ NPs/CB was lower than the Pt NPs/CB. The difference between the ORR activity obtained from DFT and experimental measurements can be due to the difference in the number of oxygen adsorption sites of these nanocatalysts.

The size of the nanoparticles is another factor that can play an important role in catalytic activity [68,69]. Therefore, Cheng et al. applied an efficient technique named ligand-assisted preparation to control the size of the PdCu nanoparticles [63]. They synthesized PdCu nanoparticles with four different sizes of 9.3, 7.1, 6.3 and 5.8 nm. It was found, via electrochemical measurements, that when the size of PdCu NPs/C decreased, the mass activity of PdCu nanoparticles increased. In addition, all bimetallic nanocatalysts showed greater catalytic activity than Pd/C nanocatalyst. To understand the size dependence on ORR catalytic activity for the PdCu nanoparticles, the AEO was calculated on icosahedral PdCu nanoparticles with sizes of 0.5, 1.1, 1.6 and 2.1 nm. Consequently, it can be inferred, based on DFT calculations, that as the particle size increases, the AEO and charge distribution of nanoparticles are closer to the bulk phase, which explains the decrease in the catalytic activity for the ORR.

**Figure 2:**
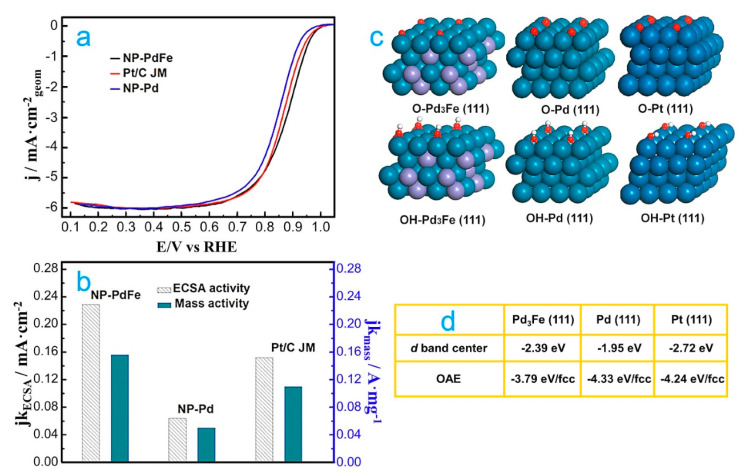
(**a**) Polarization curves for the ORR on NP-PdFe, NP-Pd and Pt/C nanocatalysts in O_2_-saturated 0.1 M HClO_4_ solution at room temperature, using 1600 rpm. (**b**) ECSA- and mass-normalized kinetic activities for all nanocatalysts at 0.9 V. (**c**) The most stable adsorbed schematic model of O atoms adsorbed on Pd_3_Fe (111), Pd (111) and Pt (111) slabs. (**d**) The corresponding OAE and *d*-band centers of Pd_3_Fe (111), Pd (111) and Pt (111) slabs. Republished with permission of Elsevier from [71].

**Figure 3:**
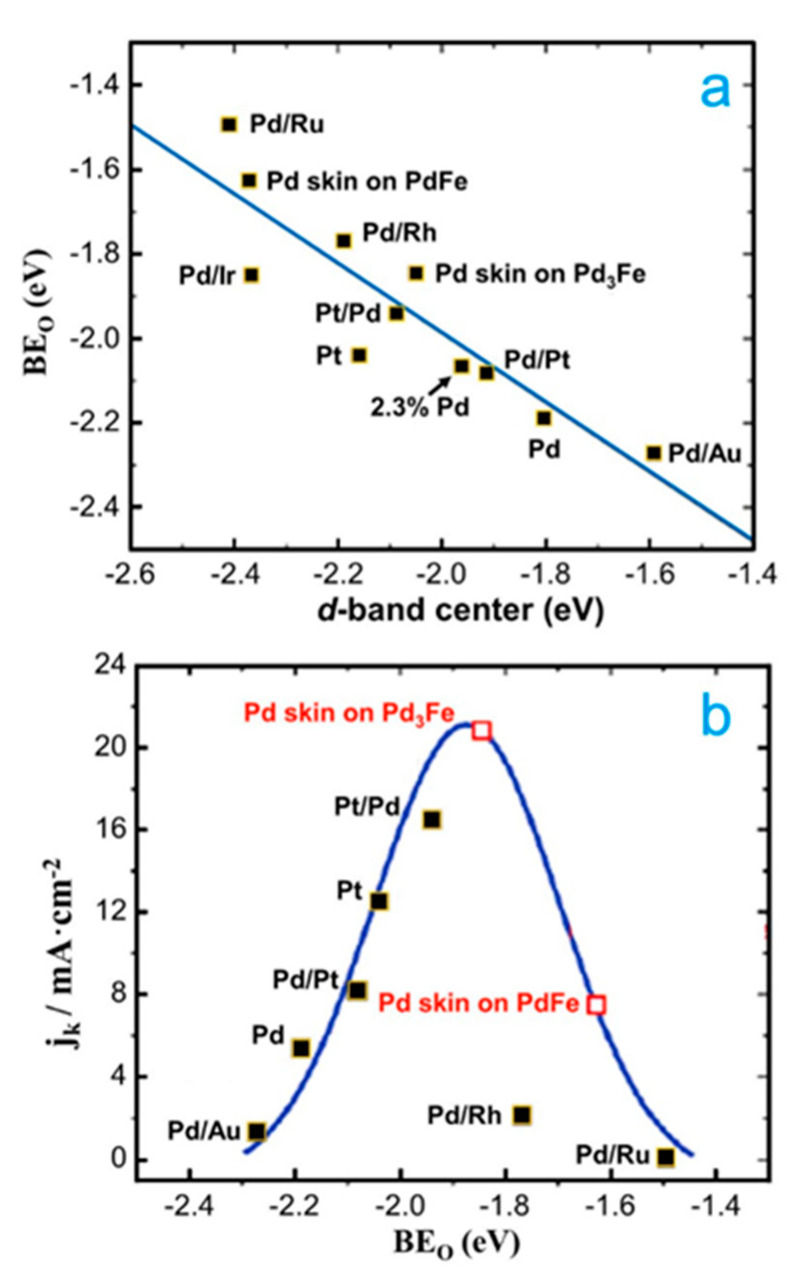
(**a**) Calculated oxygen-binding energies (BE_o_) of Pd and Pt overlayers on various substrates as a function of the Pd *d*-band center. The energies plotted correspond to the most stable configuration of O adsorption at the Pd or Pt hollow sites on each surface. (**b**) Volcano dependence of the ORR activity (expressed as the kinetic current density at 0.8 V vs. RHE, at room temperature, using a rotation rate of 1600 rpm) as a function of the calculated oxygen-binding energy. Note that the experimental data and the predicted current densities of the Pd overlayer on Pd_3_Fe (111) and PdFe (111) surfaces, based on the calculated oxygen-binding energy, are shown as the solid and open squares, respectively. Republished with permission of American Chemical Society from [72].

In addition to all the research on Pd-Cu alloys for the ORR in acid media, there are other studies combining theory and experiments related to 3*d* transition metals (e.g., Co, Fe and Ti), which were also alloyed with Pd due to the cost of the first ones [70,71,72,73]. For example, Han and co-workers prepared a PdFe nanoporous (NP-PdFe) alloy by dealloying it from its PdFeAl precursor [71]. The authors determined that NP-PdFe exhibits superior catalytic activity (Figure 2a,b) and stability for the ORR in comparison to that of both NP-Pd and Pt/C. To explain the origin of the enhanced ORR catalytic activity of NP-PdFe, the *d*-band centers and the adsorption energy of reaction intermediates (O and OH) were computed (Figure 2c). The *d*-band center of Pd in PdFe is shifted to a lower value with respect to the pure Pd after being alloyed with Fe (Figure 2d). In addition, the adsorption energies of ORR intermediates showed a lower value for PdFe (111) than Pd (111), as shown in Figure 2d. These results show the possible origin for the higher ORR activity of NP-PdFe relative to NP-Pd. In the same way, Shao et al. also worked with Pd-Fe-ordered systems [72]. DFT calculations demonstrated a linear correlation between the *d*-band center of Pd and the oxygen adsorption (Figure 3a). In addition, a volcano-like relationship was found between the kinetic current and the oxygen-binding energy for Pd monolayers on different single-crystal surfaces. Figure 3b shows that the Pd skin on Pd_3_Fe is the best nanocatalyst for the ORR.

Another proposal was made by Liu et al. [73]; they worked with the PdTiAl precursor to obtain PdTi nanoporous (NP-PdTi) nanocatalysts. The authors determined that the NP-PdTi presented a superior catalytic activity and stability for the ORR in comparison to that of NP-Pd and Pt/C. DFT computations were further used to investigate the possible origin of the enhanced ORR activity after alloying Ti with Pd. These computations showed that the *d*-band center values of Pd in PdTi (−2.67 eV) are like that of Pt (−2.72 eV), but the *d*-band center of Pd in PdTi is abruptly shifted to a lower value with respect to pure Pd (−1.95 eV). In addition, the OAE on the PdTi (111) surface was lower than on Pd (111) and Pt (111) surfaces. Consequently, it can be inferred that the modification of the electronic properties of PdTi produces a decrement in the OAE, which can be the reason for the improved catalytic activity of PdTi.

**Figure 4:**
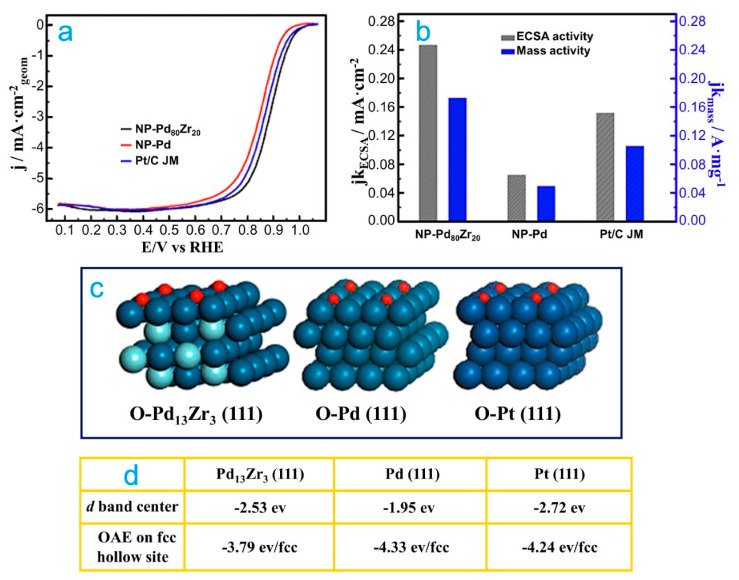
(**a**) Polarization curves for the ORR on NP-Pd_80_Zr_20_, NP-Pd and Pt/C nanocatalysts in the O_2_-saturated 0.1 M HClO_4_ solution at room temperature, using 1600 rpm. (**b**) Specific kinetic and mass kinetic current densities at room temperature for all nanocatalysts at 0.9 V. (**c**) The most stable adsorbed schematic model of O atoms adsorbed on Pd_13_Zr_3_ (111), Pd (111) and Pt (111) slabs. (**d**) The corresponding *d*-band center and OAE. Republished with permission of Elsevier from [74].

In addition to all the studies on 3*d* transition metal-Pd alloys for the ORR in acid media, there are other ones that not only combine theory and experiments on 4*d* transition metals, but also incorporate rare earth materials alloyed with Pd (e.g., Zr [74], Y [75] and Ce [76]). For example, for the Zr-Pd nanocatalyst, Duan et al. obtained PdZr nanoporous (NP-PdZr) alloy [74], which was fabricated by dealloying it from the PdZrAl precursor alloy. The NP-Pd_80_Ti_20_ nanocatalyst presented a superior catalytic activity and stability for the ORR with respect to that of NP-Pd and Pt/C (Figure 4a,b). To explain the origin of catalytic activity of NP-Pd_80_Ti_20_ nanocatalyst, the *d*-band center and OAE were computed on Pd_13_Zr_3_ (111) alloy (Figure 4c,d). It was demonstrated that the *d*-band center of Pd in PdZr (−2.53 eV) is like that of Pt (−2.72 eV), but a negative shift occurs with respect to pure Pd (−1.95 eV). In addition, the OAE on Pd_13_Zr_3_ (111) was lower than that on Pd (111) and Pt (111) surfaces. Consequently, once more, it can be inferred that the modification of the electronic properties of NP-PdZr produces a decrease in the OAE, which can be the reason for the improved catalytic activity of NP-PdZr for the ORR in acid media.

**Figure 5:**
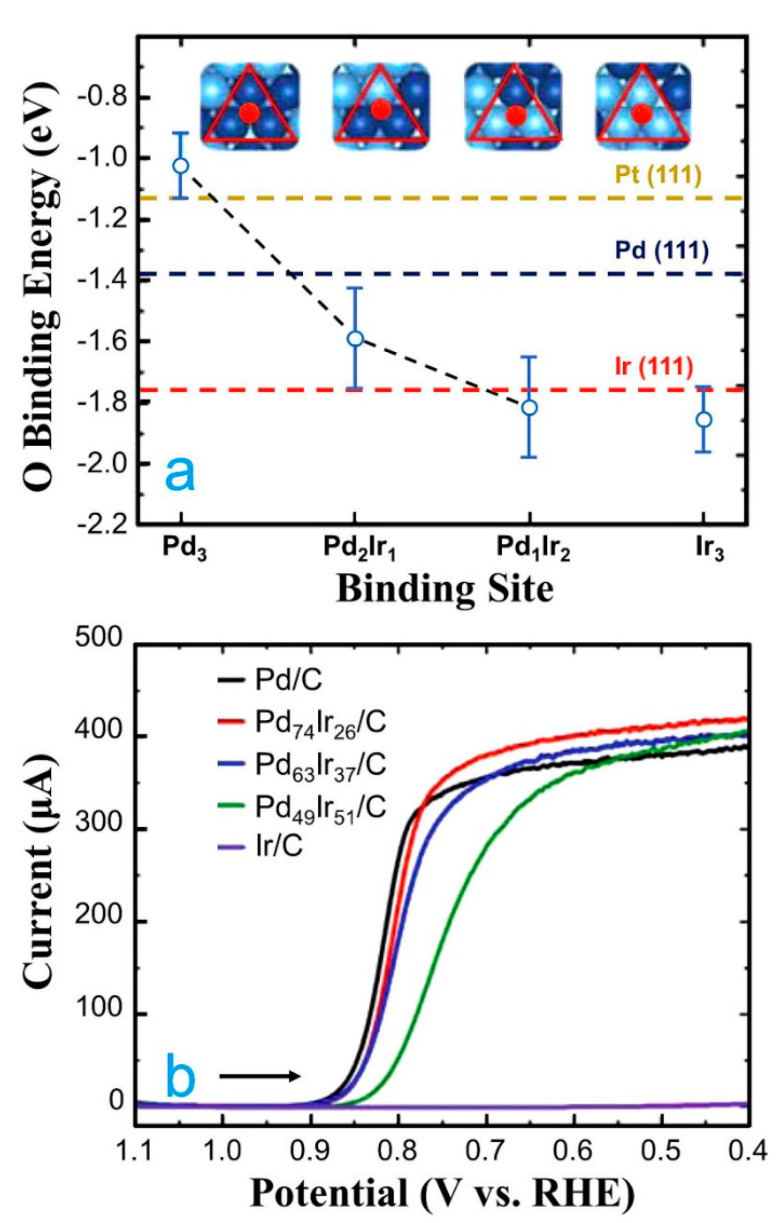
(**a**) Plot of the calculated O-binding energies for the four different triatomic ensembles of PdIr on the randomly alloyed Pd_50_Ir_50_ (111) model. (**b**) Rotating disk voltammograms (RDVs) showing the activity of all nanocatalysts towards the ORR in O_2_-saturated, 0.10 M HClO_4_. The scans commenced at 1.10 V and continued to 0.40 V (vs. RHE), using a scan rate of 20.0 mV s^−1^ and a rotation rate of the electrode of 1600 rpm. Note that arrows in both frames indicate the scan direction. Republished with permission of Royal Society of Chemistry from [80].

Another strategy that is used to improve the catalytic activity and stability of Pd-based nanocatalysts is forming alloys of Pd with other noble metals [77,78,79]. This strategy has gained great importance since noble metals are less prone to degradation problems in comparison to 3*d* transition metals. In this direction, there are some theoretical and experimental studies where Pd was alloyed with noble metals for the ORR in acid media [80,81]. For example, DFT calculations were used as a predictive tool to investigate the catalytic activity of PdIr alloys [80]. Four different triatomic ensembles of PdIr on randomly alloyed Pd_50_Ir_50_ (111) surfaces were analyzed in detail. The O-binding energies of the triatomic ensembles increase as the Ir content tends to increase (Figure 5a). It suggests that increasing the Ir content can reduce the catalytic activity for the ORR. The theoretical data obtained were corroborated by the experimental data (Figure 5b). In another study, PdIr nanoparticles were evaluated for the ORR combining experimental procedures and theoretical data [81]. The Pd_5_Ir/C and Pd_5_Ir_3_/C nanocatalysts presented higher catalytic activity than Pd/C. In addition, Pd_5_Ir/C was the most stable nanocatalyst in comparison to Pd/C and Pt/C after 3000 h of operation in a membrane-electrode assembly. To understand the good catalytic activity and stability of PtIr/C nanocatalysts, DFT calculations were done on Pd/Pd/Pd_3_Ir (111) and Pd (111) surfaces. The OAE on the Pd/Pd/Pd_3_Ir (111) surface was 0.1 eV lower than Pd (111). Based on theoretical insights and experimental findings, one can infer that the enhanced ORR activity of PdIr nanocatalysts can be attributed to the decrease of the OAE on Pd in the presence of Ir.

**Figure 6:**
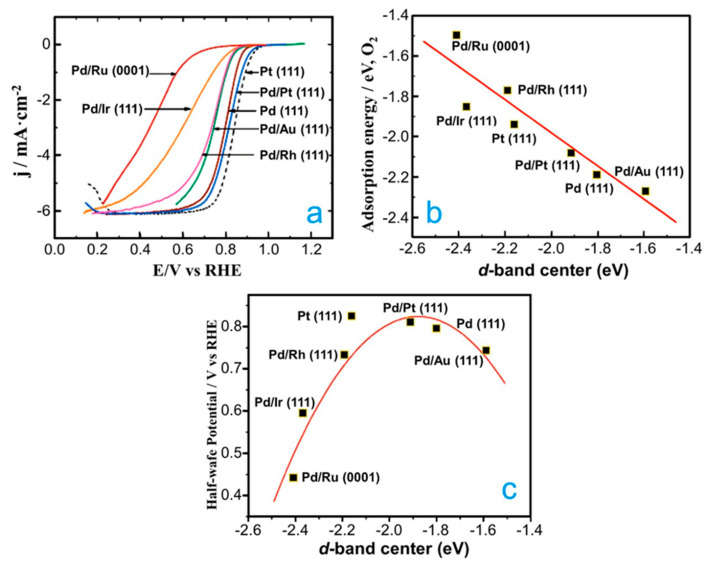
(**a**) Comparison of polarization curves for the ORR on Pd monolayers on different substrates and on Pd (111) and Pt (111) in a 0.1 M HClO_4_ solution. Sweep rate of 10 mV s^−1^ at room temperature. (**b**) Calculated O_2_ adsorption energies on Pd monolayers on various substrates and (**c**) half-wave potentials for the ORR on Pd monolayers on different substrates in a 0.1 M HClO_4_ solution, both as a function of the calculated Pd *d*-band center (relative to the Fermi level). Republished with permission of American Chemical Society from [82].

Finally, there are other studies that compare the catalytic activities and stabilities of Pd alloyed with different elements [82,83]. For example, the catalytic activities of Pd monolayers deposited on Ru (0001), Rh (111), Ir (111), Pt (111) and Au (111) for the ORR were investigated using DFT calculations and experimental procedures [82]. The catalytic activities showed the following trend: Pd/Pt (111) > Pd/Au (111) > Pd/Rh (111) > Pd/Ir (111) > Pd/Ru (0001) (Figure 6a). In addition, a linear correlation between the oxygen-binding energies and the *d*-band position of the Pd monolayers on various structures was computed (Figure 6b). Interestingly, when the measured half-wave potentials were plotted vs. the computed *d*-band center, a volcano-type trend was observed (Figure 6c). In a more recent study, the catalytic activity of Pd_3_-M (M = Ag, Co and Fe) nanoparticles supported on graphene nanosheets (Pd_3_-M/GNS) was investigated for the ORR [83]. The catalytic activities of the Pd_3_Fe/GNS (1.26 mA cm^−2^) and Pd_3_Co/GNS (0.93 mA cm^−2^) nanocatalysts were higher than Pd/GNS (0.75 mA cm^−2^) at the potential of 0.85 V vs. RHE, whereas that Pd_3_Ag/GNS nanocatalyst (0.10 mA cm^−2^) presented a very low catalytic activity. To understand the catalytic activity trend of the Pd_3_-M nanocatalysts, the *d*-band center was computed using DFT calculations for these systems. The computed *d*-band center of Pd_3_Ag, Pd_3_Fe and Pd_3_Co alloys were −1.69, −2.21 and −2.15 eV, respectively. The *d*-band centers of Pd_3_Fe and Pd_3_Co were downward shifted in comparison to pure Pd, while for Pd_3_Ag, it was upward shifted compared to pure Pd. Consequently, it can be inferred that the improved catalytic activity of PdFe and PdCo can be attributed to the modifications of the electronic properties of Pd due to the presence of 3*d* metals in the alloys.

### 3.2. Ir-Based Bimetallic Nanocatalysts

Another alternative to design Pt-free nanocatalysts for the ORR is employing Ir-based nanocatalysts [84,85]. Currently, it is reported in the literature that Ir-based nanocatalysts have been analyzed combining theory and experiment [86]. First, catalytic activity, stability and durability of different Ir_3_M (111), Ir monolayers supported on the Ir_3_M (111) surface and Ir bilayers on the surface of Ir_3_M (111) (M = 3*d*, 4*d* and 5*d* transition metal) were investigated. Based on OAE, thermodynamic properties and aggregation energies, the Ir monolayer supported on the Ir_3_Cr (111) surface was the best candidate from a computational point of view. After that, the computational results were experimentally validated. The catalytic activity of the Ir skin layer/Ir_3_Cr nanocatalyst (1.180 mA cm^−2^) was much higher than pure Ir (0.096 mA cm^−2^) measured at 0.7 V vs. RHE. In addition, the Ir skin layer/Ir_3_Cr nanocatalyst presented good stability in ORR condition, which agrees with the DFT results.

## 4. Pt-Free Trimetallic Nanocatalysts

In the last decade, to improve the catalytic activity and stability of the Pt-free nanocatalysts for the ORR in acid media, Pt-free trimetallic nanocatalysts have been computationally designed, which can present higher catalytic activities and stability than bimetallic nanocatalysts [87,88,89]. Although the theoretical and experimental studies for the investigation of novel nanocatalysts are scarce, there are certainly some couple of studies in this direction. For example, the PdCoIr nanocatalyst was investigated for the ORR in acid media combining theory and experiment [90]. At the DFT level, it was demonstrated that the out-diffusion of Co atoms can be suppressed due to the presence of Ir atoms in the sub-surface layers, suggesting good stability for PdCoIr systems. In addition, the O and OH adsorption energies on the PdCoIr surface were lower than that on the Pd surfaces, which can be attributed to the modification of the surface-electronic structure of the PdCoIr system. Finally, it was found that PdCoIr system causes a decrease in the activation barriers of O/OH hydrogenation reactions compared to pure Pd. From these theoretical results, it can be inferred that the PdCoIr system is a good candidate for the ORR. Consequently, in order to validate the theoretical insights, Pd_3_CoIr_0.6_/C, Pd_3_Co/C and Pd/C were experimentally evaluated for the ORR in acid media. The electrochemical results agreed with the theoretical predictions, since the maximum catalytic activity was for the trimetallic nanocatalyst.

## 5. Conclusions and Outlook

The design of highly efficient nanocatalysts for the ORR is key to achieve the massive use of PEMFCs. In this direction, the Pt-free metal nanocatalysts have received considerable relevance due to their good catalytic activity and slightly lower cost with respect to Pt. Consequently, coupled studies, combining experimental findings and theoretical insights, have allowed for outstanding advances in the design of novel Pt-free metal nanocatalysts for the ORR. Based on this review, the following conclusions and future directions can be proposed.

Numerous Pt-free nanocatalysts have been designed for the ORR in acid media combining theory and experiment. However, at the DFT level, the nanocatalysts have been studied considering several predictors, i.e., the adsorption energy of the reaction intermediates and the *d*-band center. Therefore, in order to have a wider overview at the DFT level, we consider important to calculate the free energy diagrams to define the rate-determining step of the ORR mechanism.

To date, combined studies on Pt-free trimetallic nanocatalysts are very scarce. Therefore, we consider it as a great opportunity to design highly efficient nanocatalysts for the ORR. It is worth noting that the design of this kind of nanocatalyst has proved fruitful when theory and experiments are combined.

It has been documented that the novel catalyst support materials (e.g., 1D and 2D structures) play a very important role on the catalytic activity toward the ORR in acid media. However, in the combined studies on Pt-free metal nanocatalysts for the ORR, the investigations that analyze the effect of the support material on the catalytic activity are very scarce. Therefore, it is necessary to develop combined investigations to understand the effect of the support material on the catalytic activity and stability of Pt-free metal nanocatalysts toward the ORR.

In the coupled studies, DFT calculations are mainly developed using the generalized-gradient approximation. Hence, it is important to employ more sophisticated methodologies (e.g., hybrid functionals or dispersion corrections) to improve the description of the interaction between ORR intermediaries and metal surfaces.

## Data Availability

Not applicable.
